# (*E*)-4-Meth­oxy-2-(*o*-tolyl­imino­meth­yl)phenol

**DOI:** 10.1107/S1600536809009192

**Published:** 2009-03-19

**Authors:** Arzu Özek, Orhan Büyükgüngör, Çiğdem Albayrak, Mustafa Odabaşoğlu

**Affiliations:** a Department of Physics, Ondokuz Mayıs University, TR-55139 Samsun, Turkey; b Sinop University, Sinop Faculty of Education, Sinop, Turkey; c Pamukkale University, Denizli Technical Vocational School, Denizli, Turkey

## Abstract

In the mol­ecule of the title compound, C_15_H_15_NO_2_, the aromatic rings are oriented at a dihedral angle of 15.46 (6)°. An intra­molecular O—H⋯N hydrogen bond results in the formation of a nearly planar six-membered ring [maximum deviation of 0.035 (5) Å for the N atom] which is almost coplanar with the adjacent ring, making a dihedral angle of 0.8 (3)°. The title organic mol­ecule is a phenol–imine tautomer, as evidenced by the C—O, C—N and C—C bond lengths. Mol­ecules are linked by inter­molecular C—H⋯O hydrogen bonds that generate a *C*(5) chain. C—H⋯π and π–π inter­actions exist in the structure. The π–π inter­action occurs between the phenol ring and its symmetry equivalent at (1 − *x*, 1 − *y*, −*z*), with a centroid–centroid distance of 3.727 (7) Å and a plane-to-plane separation of 3.383 (5) Å, resulting in an offset angle of 24.82 (1)°.

## Related literature

For previous work in our structural study of Schiff bases, see: Özek *et al.* (2007 ▶); Odabaşoğlu, Arslan *et al.* (2007 ▶); Odabaşoğlu, Büyükgüngör *et al.* (2007 ▶). For a related compound, see: Albayrak *et al.* (2005 ▶). 
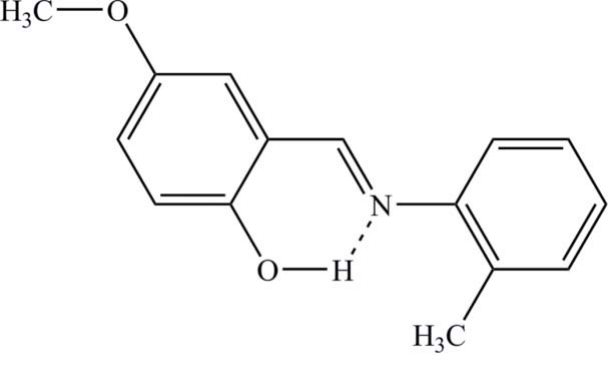


## Experimental

### 

#### Crystal data

C_15_H_15_NO_2_
*M* *_r_* = 241.28
Monoclinic, 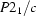

*a* = 13.2889 (6) Å
*b* = 8.5986 (6) Å
*c* = 11.6714 (6) Å
β = 113.284 (3)°
*V* = 1225.03 (12) Å^3^
*Z* = 4
Mo *K*α radiation
μ = 0.09 mm^−1^
*T* = 100 K
0.59 × 0.47 × 0.30 mm

#### Data collection

Stoe IPDS II diffractometer
Absorption correction: integration (*X-RED32*; Stoe & Cie, 2002 ▶) *T*_min_ = 0.954, *T*_max_ = 0.975
6569 measured reflections
2537 independent reflections
2108 reflections with *I* > 2σ(*I*)
*R*_int_ = 0.039

#### Refinement

*R*[*F* ^2^ > 2σ(*F* ^2^)] = 0.036
*wR*(*F* ^2^) = 0.092
*S* = 1.02
2537 reflections
223 parameters
H atoms treated by a mixture of independent and constrained refinement
Δρ_max_ = 0.20 e Å^−3^
Δρ_min_ = −0.19 e Å^−3^

### 

Data collection: *X-AREA* (Stoe & Cie, 2002 ▶); cell refinement: *X-AREA*; data reduction: *X-RED32* (Stoe & Cie, 2002 ▶); program(s) used to solve structure: *SHELXS97* (Sheldrick, 2008 ▶); program(s) used to refine structure: *SHELXL97* (Sheldrick, 2008 ▶); molecular graphics: *ORTEP-3 for Windows* (Farrugia, 1997 ▶); software used to prepare material for publication: *WinGX* (Farrugia, 1999 ▶).

## Supplementary Material

Crystal structure: contains datablocks I, global. DOI: 10.1107/S1600536809009192/bv2115sup1.cif

Structure factors: contains datablocks I. DOI: 10.1107/S1600536809009192/bv2115Isup2.hkl

Additional supplementary materials:  crystallographic information; 3D view; checkCIF report

## Figures and Tables

**Table 1:** Hydrogen-bond geometry (Å, °) *Cg*2 is the centroid of the  C9–C14 ring.

*D*—H⋯*A*	*D*—H	H⋯*A*	*D*⋯*A*	*D*—H⋯*A*
O1—H1⋯N1	0.958 (19)	1.68 (2)	2.5794 (13)	154.8 (17)
C8—H8⋯O1^i^	0.973 (15)	2.444 (15)	3.4129 (14)	173.5 (12)
C7—H7*B*⋯*Cg*2^ii^	1.01 (2)	2.90 (2)	3.6727 (16)	134 (2)

Symmetry codes: (i) 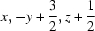; (ii) 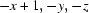.

**Fig. 1.**
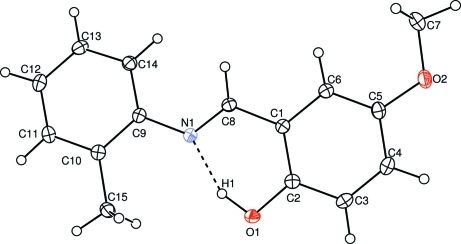
A view of (I), with the atom-numbering scheme and 30% probability displacement ellipsoids. Dashed line indicates intramolecular hydrogen bond.

**Fig. 2.**
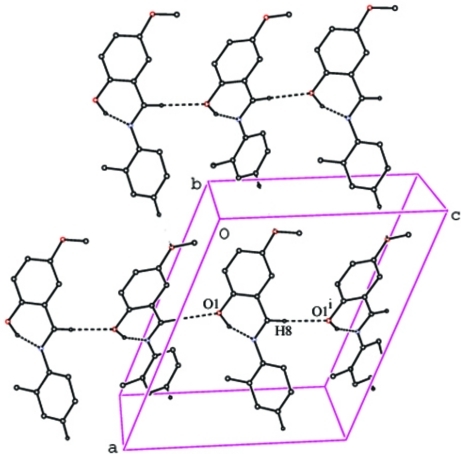
A partial packing view of (I), showing the formation of the C(5) chain through C—H···O intermolecular hydrogen bonds. Dashed lines indicate hydrogen bonds. H atoms not involved in hydrogen bonding have been omitted for clarity. [Symmetry code: (i) x, -y + 3/2, z + 1/2].

**Fig. 3.**
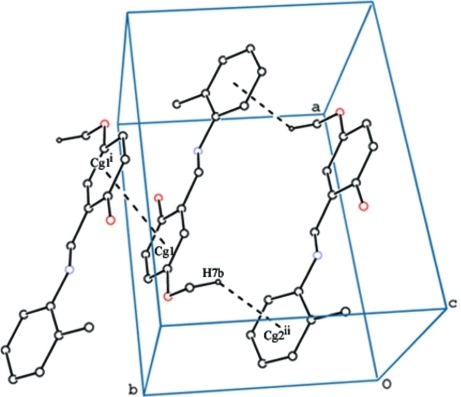
A partial packing view of (I), showing the intermolecular C—H···π and π···π interactions as dashed lines. H atoms not involved in hydrogen bonding have been omitted for clarity. [Symmetry codes; (i): 1 - x, -y + 2, 1 - z; (ii): 1 - x, 1 - y, 1 - z].

## supplementary crystallographic information

### Comment

The present work is part of a structural study of Schiff bases (Özek *et al.*, 2007; Odabaşoğlu, Büyükgüngör *et al.*, 2007; Odabaşoğlu, Arslan *et al.*, 2007) and we report here the structure of (*E*)-4-methoxy-2-[(*o*-tolylimino)methyl]phenol, (I).

In general, *o*-hydroxy Schiff bases exhibit two possible tautomeric forms, the phenol-imine (or benzenoid) and keto-amine (or quinoid) forms. Depending on the tautomers, two types of intra-molecular hydrogen bonds are possible: O—H···N in benzenoid and N—H···O in quinoid tautomers. The H atom in title compound (I) is located on atom O1, thus the phenol-imine tautomer is favored over the keto-amine form, as indicated by the C2—O1 [1.3579 (2) Å], C8—N1 [1.2865 (2) Å], C1—C8 [1.4519 (2) Å] and C1—C2 [1.4071 (2) Å] bond lengths (Fig. 1). The C2—O1 bond length of 1.357 (2)Å indicates single-bond character, whereas the C8—N1 bond length of 1.286 (2)Å indicates a high degree of double-bond character. Similar results were observed for 2-(3-methoxysalicylideneamino)-1*H*-benzimidazolemonohydrate [C—O=1.357 (2) Å, C—N= 1.285 (2) Å, Albayrak *et al.*, 2005].

It is known that Schiff bases may exhibit thermochromism or photochromism, depending on the planarity or non-planarity of the molecule, respectively. Therefore, one can expect photochromic properties in (I) caused by non-planarity of the molecules; the dihedral angle between rings A(C1—C6) and B (C9—C14) is 15.46 (6)°. The intramolecular O—H···N hydrogen bond (Table 1) results in the formation of a nearly planar six-membered ring C (O1/H1/N1/C1/C2/C8), oriented with respect to rings A and B at dihedral angles of A/C=0.81 (2)° and B/C= 15.04 (3)°. So, it is coplanar with the adjacent ring A and generates an S(6) ring motif. The O1···N1 distance of 2.579 (2)Å is comparable to those observed for analogous hydrogen bonds in three (*E*)-2-[(bromophenyl)iminomethyl]-4-methoxyphenols [2.603 (2) Å, 2.638 (7) Å, 2.577 (4) Å; Özek *et al.*, 2007]. In the crystal structure, weak intermolecular C—H···O hydrogen bonds results in the formation of C(5) chains along the *c* axis (Table 1, Fig. 2). In addition to these intermolecular interactions, C—H···π interactions and π···π interactions play roles in the crystal packing (Table 1, Fig. 3). This slipped π···π interaction occurs between *Cg*1 (the centroid of the C1—C6 ring) and its symmetry equivalent at (1 - *x*, 1 - *y*, -*z*), with a centroid-to-centroid distance of 3.727 (7)Å and a plane-to-plane separation of 3.383 (5) Å, resulting in an offset angle of 24.82 (1)°.

### Experimental

The compound (*E*)-4-methoxy-2-[(*o*-tolylimino)methyl]phenol was prepared by reflux a mixture of a solution containing 5-methoxysalicylaldehyde (0.5 g 3.3 mmol) in 20 ml ethanol and a solution containing 2-chloraniline (0.420 g 3.3 mmol) in 20 ml ethanol. The reaction mixture was stirred for 1 h under reflux. The crystals of (*E*)-4-methoxy-2-[(*o*-tolylimino)methyl]phenol suitable for X-ray analysis were obtained from methanol by slow evaporation (yield % 73; m.p. 343–344 K).

### Refinement

All the H-atoms were found in difference-density maps, and refined freely. The C—H bond lengths are 0.95 (2)–0.99 (2) Å.

### Crystal data

**Table:**

C_15_H_15_NO_2_	*F*(000) = 512
*M**_r_* = 241.28	*D*_x_ = 1.308 Mg m^−^^3^
Monoclinic, *P*2_1_/*c*	Mo *K*α radiation, λ = 0.71073 Å
Hall symbol: -P 2ybc	Cell parameters from 6569 reflections
*a* = 13.2889 (6) Å	θ = 1.9–28.0°
*b* = 8.5986 (6) Å	µ = 0.09 mm^−^^1^
*c* = 11.6714 (6) Å	*T* = 100 K
β = 113.284 (3)°	Prism, red
*V* = 1225.03 (12) Å^3^	0.59 × 0.47 × 0.30 mm
*Z* = 4	

### Data collection

**Table:**

Stoe IPDS II diffractometer	2537 independent reflections
Radiation source: fine-focus sealed tube	2108 reflections with *I* > 2σ(*I*)
plane graphite	*R*_int_ = 0.039
Detector resolution: 6.67 pixels mm^-1^	θ_max_ = 26.5°, θ_min_ = 2.9°
ω scans	*h* = −16→16
Absorption correction: integration (*X-RED32*; Stoe & Cie, 2002)	*k* = −9→10
*T*_min_ = 0.954, *T*_max_ = 0.975	*l* = −14→13
6569 measured reflections	

### Refinement

**Table:**

Refinement on *F*^2^	Primary atom site location: structure-invariant direct methods
Least-squares matrix: full	Secondary atom site location: difference Fourier map
*R*[*F*^2^ > 2σ(*F*^2^)] = 0.036	Hydrogen site location: inferred from neighbouring sites
*wR*(*F*^2^) = 0.092	H atoms treated by a mixture of independent and constrained refinement
*S* = 1.02	*w* = 1/[σ^2^(*F*_o_^2^) + (0.0457*P*)^2^ + 0.2485*P*] where *P* = (*F*_o_^2^ + 2*F*_c_^2^)/3
2537 reflections	(Δ/σ)_max_ < 0.001
223 parameters	Δρ_max_ = 0.20 e Å^−^^3^
0 restraints	Δρ_min_ = −0.19 e Å^−^^3^

### Special details

**Table:**

Experimental. 133 frames, detector distance = 80 mm
Geometry. All e.s.d.'s (except the e.s.d. in the dihedral angle between two l.s. planes) are estimated using the full covariance matrix. The cell e.s.d.'s are taken into account individually in the estimation of e.s.d.'s in distances, angles and torsion angles; correlations between e.s.d.'s in cell parameters are only used when they are defined by crystal symmetry. An approximate (isotropic) treatment of cell e.s.d.'s is used for estimating e.s.d.'s involving l.s. planes.
Refinement. Refinement of *F*^2^ against ALL reflections. The weighted *R*-factor *wR* and goodness of fit *S* are based on *F*^2^, conventional *R*-factors *R* are based on *F*, with *F* set to zero for negative *F*^2^. The threshold expression of *F*^2^ > σ(*F*^2^) is used only for calculating *R*-factors(gt) *etc*. and is not relevant to the choice of reflections for refinement. *R*-factors based on *F*^2^ are statistically about twice as large as those based on *F*, and *R*- factors based on ALL data will be even larger.

### Fractional atomic coordinates and isotropic or equivalent isotropic displacement parameters (Å^2^)

**Table:**

	*x*	*y*	*z*	*U*_iso_*/*U*_eq_	
C1	0.50912 (9)	0.80474 (14)	0.44057 (10)	0.0202 (2)	
C2	0.50739 (9)	0.87880 (14)	0.33229 (10)	0.0220 (3)	
C3	0.41277 (10)	0.95544 (15)	0.25458 (11)	0.0260 (3)	
C4	0.32140 (10)	0.95800 (15)	0.28313 (11)	0.0268 (3)	
C5	0.32211 (9)	0.88566 (14)	0.39055 (11)	0.0240 (3)	
C6	0.41552 (9)	0.80964 (14)	0.46900 (11)	0.0221 (3)	
C7	0.22417 (11)	0.82076 (17)	0.51644 (13)	0.0301 (3)	
C8	0.60665 (9)	0.72552 (14)	0.52526 (10)	0.0204 (2)	
C9	0.78609 (9)	0.62921 (13)	0.57661 (10)	0.0199 (2)	
C10	0.88252 (9)	0.65581 (14)	0.55698 (10)	0.0220 (3)	
C11	0.97626 (10)	0.57301 (15)	0.62954 (11)	0.0253 (3)	
C12	0.97591 (10)	0.46596 (15)	0.71788 (11)	0.0273 (3)	
C13	0.87983 (10)	0.43903 (15)	0.73477 (11)	0.0267 (3)	
C14	0.78528 (10)	0.52031 (15)	0.66497 (11)	0.0238 (3)	
C15	0.88540 (10)	0.77174 (16)	0.46210 (13)	0.0271 (3)	
N1	0.69160 (8)	0.71244 (11)	0.49897 (9)	0.0201 (2)	
O1	0.59622 (7)	0.88000 (11)	0.30225 (8)	0.0259 (2)	
O2	0.22688 (7)	0.89755 (11)	0.40973 (9)	0.0299 (2)	
H1	0.6488 (15)	0.821 (2)	0.3689 (18)	0.052 (5)*	
H3	0.4132 (12)	1.0039 (19)	0.1796 (14)	0.034 (4)*	
H4	0.2560 (12)	1.0104 (19)	0.2304 (14)	0.034 (4)*	
H6	0.4193 (11)	0.7593 (18)	0.5449 (14)	0.027 (3)*	
H7A	0.2795 (12)	0.8655 (18)	0.5964 (14)	0.030 (4)*	
H7B	0.2372 (13)	0.705 (2)	0.5150 (15)	0.038 (4)*	
H8	0.6040 (11)	0.6867 (17)	0.6023 (14)	0.026 (3)*	
H11	1.0416 (12)	0.5929 (17)	0.6150 (13)	0.030 (4)*	
H12	1.0432 (12)	0.4111 (18)	0.7671 (14)	0.032 (4)*	
H13	0.8786 (12)	0.3638 (19)	0.7957 (14)	0.033 (4)*	
H14	0.7188 (12)	0.4974 (18)	0.6756 (13)	0.028 (4)*	
H15A	0.9547 (15)	0.772 (2)	0.4543 (16)	0.046 (5)*	
H15B	0.8291 (13)	0.7525 (19)	0.3784 (15)	0.036 (4)*	
H7C	0.1484 (13)	0.838 (2)	0.5114 (15)	0.039 (4)*	
H15C	0.8706 (14)	0.879 (2)	0.4830 (16)	0.048 (5)*	

### Atomic displacement parameters (Å^2^)

**Table:**

	*U*^11^	*U*^22^	*U*^33^	*U*^12^	*U*^13^	*U*^23^
C1	0.0214 (5)	0.0197 (6)	0.0185 (5)	−0.0007 (4)	0.0068 (4)	−0.0023 (4)
C2	0.0249 (5)	0.0209 (6)	0.0206 (5)	−0.0010 (5)	0.0093 (4)	−0.0025 (4)
C3	0.0313 (6)	0.0239 (6)	0.0201 (5)	0.0010 (5)	0.0072 (5)	0.0020 (5)
C4	0.0238 (6)	0.0249 (6)	0.0256 (6)	0.0033 (5)	0.0033 (5)	0.0004 (5)
C5	0.0205 (5)	0.0221 (6)	0.0287 (6)	−0.0003 (5)	0.0088 (5)	−0.0041 (5)
C6	0.0242 (6)	0.0211 (6)	0.0212 (5)	0.0006 (5)	0.0091 (4)	−0.0002 (4)
C7	0.0252 (6)	0.0313 (7)	0.0381 (7)	−0.0007 (5)	0.0172 (5)	−0.0039 (6)
C8	0.0234 (5)	0.0203 (6)	0.0179 (5)	−0.0002 (4)	0.0083 (4)	−0.0015 (4)
C9	0.0211 (5)	0.0196 (6)	0.0177 (5)	0.0012 (4)	0.0063 (4)	−0.0034 (4)
C10	0.0235 (5)	0.0193 (6)	0.0223 (5)	−0.0004 (4)	0.0083 (4)	−0.0038 (5)
C11	0.0200 (5)	0.0250 (6)	0.0297 (6)	−0.0005 (5)	0.0086 (5)	−0.0034 (5)
C12	0.0239 (6)	0.0277 (7)	0.0255 (6)	0.0053 (5)	0.0047 (5)	−0.0011 (5)
C13	0.0325 (6)	0.0258 (6)	0.0221 (6)	0.0053 (5)	0.0112 (5)	0.0036 (5)
C14	0.0248 (6)	0.0248 (6)	0.0231 (5)	0.0020 (5)	0.0110 (4)	−0.0002 (5)
C15	0.0240 (6)	0.0274 (7)	0.0326 (7)	0.0012 (5)	0.0139 (5)	0.0033 (5)
N1	0.0209 (5)	0.0197 (5)	0.0194 (4)	0.0007 (4)	0.0076 (4)	−0.0012 (4)
O1	0.0280 (4)	0.0300 (5)	0.0229 (4)	0.0028 (4)	0.0135 (3)	0.0043 (4)
O2	0.0206 (4)	0.0305 (5)	0.0391 (5)	0.0038 (4)	0.0122 (4)	0.0027 (4)

### Geometric parameters (Å, °)

**Table:**

C1—C2	1.4071 (16)	C8—H8	0.973 (15)
C1—C6	1.4091 (16)	C9—C14	1.3961 (17)
C1—C8	1.4519 (16)	C9—C10	1.4068 (16)
C2—O1	1.3579 (14)	C9—N1	1.4179 (14)
C2—C3	1.3916 (17)	C10—C11	1.3934 (16)
C3—C4	1.3805 (18)	C10—C15	1.5017 (17)
C3—H3	0.971 (16)	C11—C12	1.3836 (18)
C4—C5	1.3962 (18)	C11—H11	0.963 (16)
C4—H4	0.956 (16)	C12—C13	1.3862 (18)
C5—O2	1.3734 (15)	C12—H12	0.973 (15)
C5—C6	1.3812 (17)	C13—C14	1.3862 (17)
C6—H6	0.969 (15)	C13—H13	0.966 (16)
C7—O2	1.4226 (17)	C14—H14	0.959 (15)
C7—H7A	1.008 (15)	C15—H15A	0.960 (18)
C7—H7B	1.008 (18)	C15—H15B	0.981 (16)
C7—H7C	0.996 (17)	C15—H15C	0.99 (2)
C8—N1	1.2865 (15)	O1—H1	0.958 (19)
			
C2—C1—C6	119.70 (10)	C14—C9—C10	120.19 (10)
C2—C1—C8	121.13 (10)	C14—C9—N1	123.17 (10)
C6—C1—C8	119.16 (10)	C10—C9—N1	116.58 (10)
O1—C2—C3	118.81 (11)	C11—C10—C9	118.10 (11)
O1—C2—C1	121.88 (10)	C11—C10—C15	120.67 (11)
C3—C2—C1	119.30 (11)	C9—C10—C15	121.22 (10)
C4—C3—C2	120.30 (11)	C12—C11—C10	121.82 (11)
C4—C3—H3	122.0 (9)	C12—C11—H11	121.2 (9)
C2—C3—H3	117.7 (9)	C10—C11—H11	117.0 (9)
C3—C4—C5	121.02 (11)	C11—C12—C13	119.45 (11)
C3—C4—H4	120.9 (9)	C11—C12—H12	119.3 (9)
C5—C4—H4	118.0 (9)	C13—C12—H12	121.2 (9)
O2—C5—C6	124.87 (11)	C12—C13—C14	120.27 (12)
O2—C5—C4	115.69 (11)	C12—C13—H13	120.1 (9)
C6—C5—C4	119.43 (11)	C14—C13—H13	119.6 (9)
C5—C6—C1	120.25 (11)	C13—C14—C9	120.16 (11)
C5—C6—H6	121.7 (8)	C13—C14—H14	119.2 (9)
C1—C6—H6	118.1 (8)	C9—C14—H14	120.6 (9)
O2—C7—H7A	111.8 (9)	C10—C15—H15A	112.1 (11)
O2—C7—H7B	112.2 (9)	C10—C15—H15B	112.9 (10)
H7A—C7—H7B	108.9 (13)	H15A—C15—H15B	106.8 (14)
O2—C7—H7C	104.7 (9)	C10—C15—H15C	111.7 (10)
H7A—C7—H7C	110.4 (13)	H15A—C15—H15C	108.2 (15)
H7B—C7—H7C	108.8 (13)	H15B—C15—H15C	104.7 (14)
N1—C8—C1	120.66 (10)	C8—N1—C9	122.05 (10)
N1—C8—H8	123.1 (8)	C2—O1—H1	102.5 (11)
C1—C8—H8	116.2 (8)	C5—O2—C7	116.87 (9)
			
C6—C1—C2—O1	178.64 (11)	N1—C9—C10—C11	−178.42 (10)
C8—C1—C2—O1	−0.16 (17)	C14—C9—C10—C15	179.71 (11)
C6—C1—C2—C3	−0.23 (17)	N1—C9—C10—C15	2.50 (16)
C8—C1—C2—C3	−179.03 (11)	C9—C10—C11—C12	0.70 (18)
O1—C2—C3—C4	−179.26 (11)	C15—C10—C11—C12	179.77 (12)
C1—C2—C3—C4	−0.36 (18)	C10—C11—C12—C13	0.37 (19)
C2—C3—C4—C5	0.67 (19)	C11—C12—C13—C14	−0.92 (19)
C3—C4—C5—O2	179.29 (11)	C12—C13—C14—C9	0.40 (18)
C3—C4—C5—C6	−0.38 (19)	C10—C9—C14—C13	0.69 (17)
O2—C5—C6—C1	−179.85 (11)	N1—C9—C14—C13	177.71 (11)
C4—C5—C6—C1	−0.21 (18)	C1—C8—N1—C9	−176.83 (10)
C2—C1—C6—C5	0.51 (17)	C14—C9—N1—C8	18.82 (17)
C8—C1—C6—C5	179.34 (11)	C10—C9—N1—C8	−164.07 (11)
C2—C1—C8—N1	−4.59 (17)	C6—C5—O2—C7	−2.26 (17)
C6—C1—C8—N1	176.60 (11)	C4—C5—O2—C7	178.09 (11)
C14—C9—C10—C11	−1.22 (17)		

### Hydrogen-bond geometry (Å, °)

**Table:**

*D*—H···*A*	*D*—H	H···*A*	*D*···*A*	*D*—H···*A*
O1—H1···N1	0.958 (19)	1.68 (2)	2.5794 (13)	154.8 (17)
C8—H8···O1^i^	0.973 (15)	2.444 (15)	3.4129 (14)	173.5 (12)
C7—H7B···Cg2^ii^	1.008 (18)	2.903 (17)	3.6727 (16)	134.1 (16)

Symmetry codes: (i) *x*, −*y*+3/2, *z*+1/2; (ii) −*x*+1, −*y*, −*z*.
